# Frequency of Positive Familial Criteria in Patients with Adenocarcinoma of the Esophageal-Gastric Junction and Stomach: First Prospective Data in a Caucasian Cohort †

**DOI:** 10.3390/cancers14153590

**Published:** 2022-07-23

**Authors:** Jan Schölzchen, Christoph Treese, Peter Thuss-Patience, Alicja Mrózek, Beate Rau, Hendrik Seeliger, Dirk Hartmann, Lope Estevéz-Schwarz, Britta Siegmund, Denise Horn, Mani Nassir, Severin Daum

**Affiliations:** 1 Department for Medicine (Gastroenterology, Infectious Diseases, Rheumatology), Charité—Universitätsmedizin Berlin, Corporate Member of Freie Universität Berlin, Humboldt-Universität zu Berlin, 12203 Berlin, Germany; jan.schoelzchen@charite.de (J.S.); christoph.treese@charite.de (C.T.); britta.siegmund@charite.de (B.S.); 2 Medizinische Klinik m.S. Hämatologie—Onkologie und Tumorimmunologie, Charité–Universitätsmedizin Berlin, Corporate Member of Freie Universität Berlin, Humboldt-Universität zu Berlin, 13353 Berlin, Germany; peter.thuss@charite.de (P.T.-P.); mani.nassir@charite.de (M.N.); 3 Onkologisches Zentrum Prenzlauer Berg, Onkologische Schwerpunktpraxis, 13189 Berlin, Germany; alicja.mrozek@googlemail.com; 4 Chirurgische Klinik, Charité—Universitätsmedizin Berlin, 13353 Berlin, Germany; beate.rau@charite.de; 5 Klinik für Allgemein- Viszeral- und Gefäßchirurgie, Charité—Universitätsmedizin Berlin, 12203 Berlin, Germany; hendrik.seeliger@gmx.de; 6 Katholisches Klinikum Mainz, Klinik für Innere Medizin II, 55131 Mainz, Germany; dirk.hartmann@marienhaus.de; 7 St. Havelland Kliniken GmbH Ketziner Str. 19, Allgemein- und Viszeralchirurgie, 14641 Nauen, Germany; lope.estevezschwarz@havelland-kliniken.de; 8 Institut für Medizinische Genetik und Humangenetik, Charité—Universitätsmedizin Berlin, 13353 Berlin, Germany; denise.horn@charite.de

**Keywords:** adenocarcinoma of the esophageal–gastric junction, Clinical Cancer Registry for Brandenburg and Berlin, diffuse type gastric cancer, familial intestinal gastric cancer, gastric cancer, hereditary diffuse gastric cancer

## Abstract

**Simple Summary:**

It is well known for gastric cancer patients with subtype of diffuse histology that a proportion of patients harbour an increased familial risk. Some patients and relatives even may be detected through a genetic testing. More precise studies about the frequency of hereditary criteria in a poplation with only European ancestries for adenocarcinoma of the esophagogastric junction and stomach are missing. In current guidelines regarding genetic testing criteria not all types of stomach cancer are considered as for example patients not with subtype of diffuse histology mostly have no detectable responsible gene. The aim of the current study was to register stomach cancer patients of all different types in a certain region (Berlin, Germany) and to estimate the frequency of positive familial criteria. Patients with esophageal cancer served as comparison group as familial or hereditary background, respectively, is not significant in these patients according to current knowledge. In our study, we identified positive familial criteria in about 15% of stomach cancer patients. In regard to all different types of stomach cancer, this number almost doubled. Furthermore, one third of all registered patients in this study might have a familial or hereditary background of their disease. We therefore conclude that guidelines regarding genetic testing criteria and screening examinations should be adjusted in future.

**Abstract:**

Objectives: Current prospective studies investigating the frequency of hereditary criteria in a Caucasian population for adenocarcinoma of the esophagogastric junction (AEG) and stomach (GC) are missing. Genetic testing criteria (screening criteria) for hereditary diffuse gastric cancer (HDGC) were updated in 2020, but do not address patients with intestinal histology (familial intestinal gastric cancer FIGC). Thus, we prospectively screened patients residing in Berlin newly diagnosed with AEG or GC for hereditary criteria to gain insights into the frequency of HDGC. Methods: Prospective documentation of familial/clinical parameters in patients residing in Berlin with AEG or GC over three years was conducted. Besides HDGC criteria from 2015 and revised 2020, we also documented patients fulfilling these criteria but with intestinal type gastric cancer (FIGC). Statistical analysis was performed using X2-test. Results: One hundred fifty-three patients were finally included (92 GC; male: 50 (n.s.); 61 AEG; male: 47; *p* = 0.007). Hereditary criteria for HDGC were detected in 9/92 (9.8%) (2015 criteria) and in 14/92 (15.2%) (2020 criteria) of GC patients (AEG: 2015 criteria 3/61 (4.9%) versus 4/61 according to 2020 criteria (6.5%)). Patients fulfilling hereditary criteria but with intestinal histology (FIGC) increased from 8.7% (2015) to 14.1%, respectively (2020) (AEG: 3.2% (2015) versus 6.6% (2020)). Hereditary criteria including intestinal histology were found in 29.3% (GC) and 13.1% (AEG) (*p* = 0.03) according to the 2020 criteria. Conclusions: HDGC criteria were found in 15.2% of GC patients according to the 2020 criteria. Percentage increased to 29.3% including patients with intestinal histology among the GC group, and was 13.1% in cases with AEG. These data indicate that family history seems to be of utmost importance in GC to further detect potential hereditary genetic risks. This equally applies for patients with intestinal subtype GC.

## 1. Introduction

Gastric cancer (GC) is the third leading cause of cancer-related death worldwide and is associated with classical Hereditary Diffuse Gastric Cancer (HDGC) in about 1–3% of cases defined by clinical criteria [1]. Mutation of the CDH1 gene has been shown in classical HDGC cases in about 10–40% of patients [2,3]; in single cases, a mutation in the CTNNA gene has been described, while data on MAP3K6 are weak and controversial [4,5].

In order not to miss patients with HDGC, criteria for genetic testing have been broadened by the International Gastric Cancer Linkage Consortium [6,7]. Nevertheless, these criteria still exclude patients whose tumor histology is not of diffuse type. For this group of patients, fulfilling familial criteria with intestinal histology the classification as familial intestinal gastric cancer (FIGC) has been introduced and redefined [8]. According to data from Japan and Italy, up to 40% of patients fulfill criteria for familial GC when also intestinal type of GC is included [9,10]. The high proportion of familial criteria in the Italian study may be explained by the less strict definitions of familial association that were mainly based on occurrence of GC among direct relatives [10]. Familial intestinal gastric cancer (FIGC) remains genetically unexplained and testing/clinical criteria remain conflicting [8].

Despite being biologically similar to GC, data regarding familial association in adenocarcinoma of the esophageal–gastric junction (AEG) are still sparse. Data from the Netherlands, published in 2014, suggest familial association of about 7% of AEG and Barrett’s esophagus [11]. However, familial association was already assumed when at least one first-degree relative was also diagnosed with AEG or Barrett’s esophagus [11]. Thus, familial association may have been overestimated. Except for common risk factors such as diabetes or depression, no underlying genetic background has been described so far [12].

To date, precise prospectively acquired epidemiologic data on familial criteria in AEG and GC in a European/Caucasian population in a median risk area for gastric cancer are lacking. Thus, our aim was to prospectively collect data regarding the frequency of HDGC and FIGC criteria in GC and to characterize their clinical appearance and clinical course in a Caucasian population. We included patients with AEG as validation cohort, since almost no familial association has been reported for this tumor entity.

## 2. Methods

The frequency of criteria for HDGC and FIGC was investigated in a prospective recording of all patients, suffering from GC and AEG, residing in Berlin presenting in the participating centers from June 2015 until May 2018. Patients with AEG were included as a validation cohort (acronym EpihiB: “Epidemiology of hereditary gastric cancer in Berlin”). Expanded criteria based on the International Gastric Cancer Linkage Consortium guideline for hereditary diffuse gastric cancer (HDGC), last updated in 2020 [7], were applied and compared with classical criteria formerly published in 2015 [6] (Table 1). As it was not possible to collect precise histology of familial cases (concerning lobular breast cancer and diffuse type of gastric cancer in affected relatives), we introduced two groups: a “conservatively” estimated group including only relatives with lobular breast cancer versus a “progressively” estimated group including all patients with breast cancer irrespective of their subgroup histology assuming lobular subtype. Patients were classified as FIGC in case of fulfilling the above-mentioned HDGC criteria according to International Gastric Cancer Linkage Consortium but with intestinal histology. Data were compared with those of the *Klinisches Krebsregister für Brandenburg und Berlin (Clinical Cancer Registry for Brandenburg and Berlin* (KKRBB)) being active since 2017. However, data from *Clinical Cancer Registry for Brandenburg and Berlin* comprise only index patients but not familial tumor data. Statistical analysis was performed using Excel 2016 (Microsoft, Redmond, WA, USA) and SPSS 25 (SPSS Inc., Chicago, WA, USA). A two-tailed *p*-value < 0.05 was considered as statistically significant. The study was approved by the local ethics committee (approval no. EA4/040/15).

## 3. Results

### 3.1. Basic Patient Characteristics

**Figure 1:**
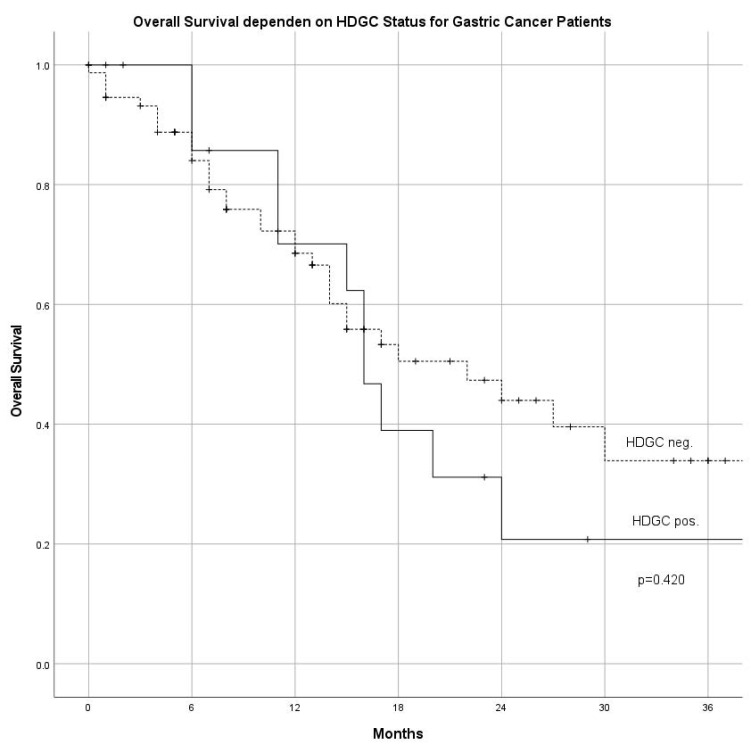
Overall survival in patients fulfilling criteria for HDGC (HDGC positive) compared with patients not fulfilling HDGC criteria (HDGC negative) (HDGC: hereditary diffuse gastric cancer).

One hundred and fifty-three patients (92 GC/61 AEG; 170 patients were screened) were included from June 2015 until May 2018. The number of male patients was higher among AEG compared with GC (f/m AEG: 14/47 vs. GC 42/50; *p* < 0.05). The overall mean age was 66.1 ± 13.8 years, in the GC-group 64.9 ± 15.0 years, and 68.0 ± 11.6 years in the AEG-group, respectively (n.s.). UICC stages at diagnosis were not different among GC and AEG subgroups (Table 1). Regarding the histological subtype according to Lauren, diffuse and mixed histology was more common in the GC group (*p* = 0.005). Association with risk factors for GC and AEG showed that *H. pylori* was found more often in patients with GC in comparison with AEG (*p* < 0.001). Association with diabetes, obesity, history of smoking, and the general incidence of cancer in the family was not different between GC and AEG (Table 1). No significant difference in overall survival was found comparing patients fulfilling HDGC criteria (HDGC pos) versus patients with diffuse type gastric cancer not fulfilling HDGC criteria (HDGC neg) (Figure 1).

### 3.2. Comparison with Data from the Clinical Cancer Registry for Brandenburg and Berlin

To validate the representation of this cohort, we compared our data with the data of the regional Clinical Cancer Registry for Brandenburg and Berlin from 2017 (Table S1). There was a significant difference regarding the mean age between both groups (EpihiB 66.1 ± 13.8 years vs. Clinical Cancer Registry for Brandenburg and Berlin 69.6 ± 12.5 years; *p* = 0.002). In detail, there were more patients aged under 60 years in the GC group of the EpihiB cohort (34.8%) compared with the registry cohort (22.0%) (*p* = 0.048). Regarding tumor localization or UICC stages, there were no significant differences between both cohorts (Table S1).

### 3.3. Familial Criteria for HDGC

“Conservative” evaluation (Table 2A) of the data detected 9 of 92 (9.8%) patients fulfilling the 2015 criteria and 14 of 92 (15.2%) patients fulfilling the 2020 HDGC criteria. In comparison with the GC group, the number of AEG patients fulfilling HDGC criteria was significantly lower (2015 criteria: 3/61 (4.9%) and 2020 criteria: 4/61 (6.6%), respectively; *p* < 0.005)). When we applied “progressive” evaluation criteria (Table 2A) assuming every breast cancer as lobular subtype, proportion of patients fulfilling HDGC criteria increased to 16.3% among patients with GC and 9.8% among patients with AEG according to the 2020 criteria (Table 2B).

Proportion of GC patients with intestinal histology fulfilling classical familial criteria (FIGC) increased similarly from 8/92 (8.7%; 2015 criteria) to 13/92 (14.1%; 2020 criteria) and was higher in comparison with the AEG group ((from 2/61 (3.2%; 2015 criteria) to 4/61 (6.6%; 2020 criteria)) (*p* < 0.007) (Table 2B).

When all patients irrespective of their primary histology (intestinal and diffuse type) were included, 13.1% (AEG) and 29.3% (GC) fulfilled the 2020 hereditary criteria. One patient with AEG aged 46 years was found to suffer from Lynch syndrome.

## 4. Discussion

In spite of new guidelines and broader application of genetic testing, current frequencies of familial criteria defining HDGC and FIGC in Caucasians are not known. With this prospective study in a well-defined region, we were able to show that 15.2% of GC patients fulfilled the expanded HDGC criteria from 2020, and that almost a third (29.3%) fulfilled the criteria when intestinal type histology was included.

Our comparison with data of the Clinical Cancer Registry for Brandenburg and Berlin showed that there is a slight bias in favor of inclusion of younger patients, which might be due to the fact that half of the participating hospitals were tertiary referral centers. KKRBB registry data provide the most recent and regional data, but the information is limited to age at tumor manifestation (<50 years) with no familial cancer history given: concerning this single criterion (age at tumor manifestation <50 years), 7% of patients from the Clinical Cancer Registry for Brandenburg and Berlin fulfilled the 2020 criteria in comparison with 14.2% among the EpihiB trial independent of histological subtype, which also supports the bias effect of tertiary referral centers in our study. However, as we were not able to acquire sufficient information about breast cancer histology, our data may underestimate patients with this familial risk constellation. Furthermore, including all types of breast cancer increased familial criteria to 35.9% in GC and 22.9% in AEG, respectively. Comparable European data collected over a broad time interval only exist from Italy. Here, occurrence of GC could be demonstrated in 18.5% of first-degree relatives of whom 70% presented with intestinal-type histology [10]. Another prospective study from Italy, conducted over three years (1985–1987), identified up to 23.9% of GC cases in first degree relatives in high-risk areas whereas in low-risk areas only 9.3% were detected [13]. In both Italian studies the increased risk of gastric cancer in first-degree relatives was either independent of histologic type [13] or even increased in cases with intestinal histology [10]. Retrospective data from a high-risk area in Tuscany (Italy) documented a rate of 33.8% GC in first- and second-degree relatives. However, only 5.9% of patients had positive familial criteria according to the 2015 International Gastric Cancer Linkage Consortium criteria, which is even slightly less in comparison with 9.8% in our study [14].

We identified almost the same percentage of patients with FIGC criteria (14.5%) as for HDGC (15.2%). We decided to adopt the HDGC criteria for FIGC in contrast to Caldas 1999 and Vogelaar for better comparison with the HDGC data [1,2]. However, data from the Italian study with index cases over a broad period (1988–2004) found even a proportion of 70% for intestinal type cancer among patients with positive familial criteria [10]. This high percentage of FIGC patients points to the problem of a missing clear genetic background in FIGC. Recently, Carvalho et al. described that patients with criteria for FIGC showed an autosomal inheritance type, more germline TP53, and other rare variants including genes such as MUTYH or MLH1 but no isolated disease causing mutation as in HDGC [8]. In the family history of our patients, there were no increased numbers of colorectal cancers, thus making an association with hereditary non-polyposis cancer unlikely (data not shown).

In AEG, 9.8% of patients (6/61) fulfilled the expanded criteria. Only one case with Lynch syndrome and AEG (fulfilling FIGC criteria) was detected. As there was a significant difference in the number of familial criteria in comparison with the GC group and as percentage of patients <50 years among the AEG group in the Clinical Cancer Registry for Brandenburg and Berlin was only 2.9%, our data do not encourage genetic background beyond, for instance, diabetes and obesity in patients with AEG. Thus, our data support the results of a Dutch registry, which found a familial clustering in 7% of AEG cases, though no genetic analysis was performed and genetic predisposition for associated risk factors such as diabetes or obesity may be responsible for part of the association [11].

## 5. Conclusions

Our current data on frequency of familial criteria in a prospective Caucasian setting support the high occurrence of familial clustering of not only HDGC but also FIGC, thus confirming the older data from high-risk areas in Italy. Further genetic deciphering in FIGC is of utmost importance. Our data from a medium risk area for gastric cancer with a high proportion of intestinal type histology prompt screening for GC in first-degree relatives for gastric cancer and at least screening for H. pylori. These points should prospectively be discussed in national guidelines.

## Figures and Tables

**Table 1:** Patient characteristics.

	Total		AEG		Gastric Cancer		*p*
	***n***	**(%)**	***n***	**(%)**	***n***	**(%)**	
Sex							
Female	56	(36.6)	14	(23.0)	42	(45.7)	**0.004**
Male	97	(63.4)	47	(77.0)	50	(54.3)	
Age							
<50	20	(13.1)	5	(8.2)	15	(16.3)	0.350
≥50	133	(86.9)	56	(91.8)	77	(83.7)	
Localization							
AEG I	29	(19.1)	29	(47.5)	X		x
AEG II	32	(21.1)	32	(52.5)	X		
Cardia	13	(8.6)	X		13	(14.1)	
Fundus	1	(0.7)	X		1	(1.1)	
Corpus	42	(27.6)	X		43	(46.7)	
Antrum	35	(23.0)	X		35	(38.0)	
UICC Stage							
I	14	(9.2)	6	(9.8)	8	(8.7)	0.860
II	23	(15)	7	(11.5)	16	(17.4)	
III	31	(20.3)	14	(23.0)	17	(18.5)	
IV	59	(38.6)	24	(39.3)	35	(38.0)	
unspecified	26	(17.0)	10	(16.4)	16	(17.4)	
Grading							
G1	8	(5.2)	4	(6.6)	4	(4.3)	0.503
G2	51	(33.3)	24	(39.3)	27	(29.3)	
G3	86	(56.2)	30	(49.2)	56	(60.9)	
unspecified	8	(5.2	3	(4.3	8	(5.4	
Lauren							
Intestinal	77	(50.3)	38	(62.3)	39	(42.4)	**0.008**
Mixed	19	(12.4)	4	(6.6)	15	(16.3)	
Diffuse	48	(31.4)	13	(21.3)	35	(38.0)	
unspecified	9	(5.9)	4	(6.6)	3	(3.3)	
Incidence of cancer in family							
Yes	88	(57.5)	20	(32.8)	31	(33.7)	0.629
No	51	(33.3)	37	(60.7)	51	(55.4)	
Unknown	14	(9.2)	4	(6.6)	10	(10.9)	
History of Smoking							
Yes	98	(64.1)	41	(67.2)	57	(62.0)	0.798
No	52	(34.0)	19	(31.1)	33	(35.9)	
Unknown	3	(2.0)	1	(1.6)	2	(2.2)	
BMI							
<25	72	(47.1)	22	(36.1)	50	(54.3)	0.39
≥25	71	(46.4)	36	(59.0)	35	(38.0)	
Unknown	10	(6.5)	3	(4.9)	7	(7.6)	
Diabetes							
Yes	33	(21.6)	15	(24.6)	18	(19.6)	0.558
No	119	(77.8)	46	(75.4)	73	(79.3)	
Unspecified	1	(0.7)	0	(0.0)	1	(1.1)	
*Helicobacter pylori* History							
Yes	29	(19.0)	3	(4.9)	29	(31.5)	**0.001**
No	51	(33.3)	22	(36.1)	26	(28.3)	
Unknown	73	(47.7)	36	(59.0)	37	(40.2)	

n: number of patients; Age: years; BMI: body mass index; significant results are depicted in bold.

**Table 2:** (**A**) Comparison of criteria from 2015 (van der Post 2015) with the 2020 updated criteria (Blair 2020). Criteria were also applied for patients with intestinal type carcinoma (AEG: adenocarcinoma of the esophageal–gastric junction; DGC: diffuse-type gastric cancer; FIGC: familial intestinal gastric cancer; HDGC: hereditary diffuse gastric cancer). (**B**) Impact to patient data according to changed criteria from 2015 (van der Post 2015) with the 2020 updated criteria (Blair 2020). Criteria were also applied for patients with intestinal-type carcinoma (FIGC); ()* inclusive unknown histology. Criteria not fulfilled by at least one patient in our cohort are not displayed in this Table (AEG: adenocarcinoma of the esophageal–gastric junction; DGC: diffuse-type gastric cancer; FIGC: familial intestinal gastric cancer; HDGC: hereditary diffuse gastric cancer).

**(A)**									
**HDGC 2015 (van der Post)**					**HDGC 2020 (Blair)**				
Family Criteria									
≥2 cases of gastric cancer in family regardless of age, with at least one DGC									
≥1 case of DGC at any age, and ≥1 case of lobular breast cancer <50 years, in different family members					≥1 case of DGC at any age, and ≥1 case of lobular breast cancer <70 years, in different family members				
≥2 cases of lobular breast cancer in family members <50 years									
Individual Criteria									
DGC <40 years					DGC <50 years				
Gastric in situ signet ring cells or pagetoid spread of signet ring cells					Gastric in situ signet ring cells or pagetoid spread of signet ring cells in individuals <50 years				
DGC at any age in individuals with a personal or family history (1st degree) of cleft lip or cleft palate									
History of DGC and lobular breast cancer, one diagnosed <50 years					History of DGC and lobular breast cancer, both diagnosed <70 years				
Bilateral lobular breast cancer, diagnosed <50 years					Bilateral lobular breast cancer, diagnosed <70 years				
FIGC 2015 (van der Post)					FIGC 2020 (Blair)				
Family Criteria									
≥2 cases of gastric cancer in family regardless of age									
≥1 case of gastric cancer at any age, and ≥1 case of lobular breast cancer <50 years, in different family members					≥1 case of gastric cancer at any age, and ≥1 case of lobular breast cancer <70 years, in different family members				
Individual Criteria									
Gastric Cancer <40 years					Gastric Cancer <50 years				
GC at any age in individuals with a personal or family history (1st degree) of cleft lip or cleft palate									
History of gastric Cancer and lobular breast cancer, one diagnosed <50 years					History of gastric cancer and lobular breast cancer, both diagnosed <70 years				
**(B)**									
	**AEG (n = 61)**		**GC (n = 92)**		**AEG (n = 61)**		**GC (n = 92)**		
**HDGC 2015 (van der Post)**					**HDGC 2020 (Blair)**				
Family Criteria	n	%	n	%	n	%	n	%	Family Criteria
≥2 cases of gastric cancer in family regardless of age, with at least one DGC	-	-	5	5.4	-	-	5	5.4	≥2 cases of gastric cancer in family regardless of age, with at least one DGC
≥1 case of DGC at any age, and ≥1 case of lobular breast cancer <50 years, in different family members	-	-	-	-	−(2) *	−(3.2) *	−(1) *	−(1.1) *	≥1 case of DGC at any age, and ≥1 case of lobular breast cancer <70 years, in different family members
Individual Criteria	n	%	n	%	n	%	n	%	Individual Criteria
DGC <40 years	3	4.9	3	3.3	4	6.5	7	7.6	DGC <50 years
History of DGC and lobular breast cancer, one diagnosed <50 years	-	-	1	1.1	-	-	2	2.2	History of DGC and lobular breast cancer, both diagnosed <70 years
**Total**	**3**	**4.9**	**9**	**9.8**	**4 (6)**	**6.5 (9.8)**	**14 (15)**	**15.2 (16.3)**	**Total**
**FIGC 2015 (van der Post)**					**FIGC 2020 (Blair)**				
Family Criteria	n	%	n	%	n	%	n	%	Family Criteria
≥2 cases of gastric cancer in family regardless of age	2	3.3	6	6.5	2	3.3	6	6.5	≥2 cases of gastric cancer in family regardless of age
≥1 case of gastric cancer at any age, and ≥1 case of lobular breast cancer <50 years, in different family members	-	-	−(2)	−(2.2)	−(4)	−(6)	−(5)	−(5.4)	≥1 case of gastric cancer at any age, and ≥1 case of lobular breast cancer <70 years, in different family members
Individual Criteria	n	%	n	%	n	%	n	%	Individual Criteria
Gastric cancer <40 years	-	-	2	2.2	2	3.3	7	7.6	Gastric cancer <50 years
**Total**	**2**	**3.3**	**8 (10)**	**8.7 (10.9)**	**4 (8)**	**6.6 (13.0)**	**13 (18)**	**14.1 (19.6)**	**Total**

## Data Availability

The data presented in this study are available on request from the corresponding author.

## Supplementary Materials

The following supporting information can be downloaded at: https://www.mdpi.com/article/10.3390/cancers14153590/s1, Table S1: Comparison of EpihiB (Epidemiology of hereditary gastric cancer in Berlin) data with data of the Clinical Cancer Registry for Brandenburg and Berlin (KKRBB) (AEG: adenocarcinoma of the esophageal–gastric junction; UICC: union internationale contre le cancer).

## Abbreviations

AEG: adenocarcinoma of the esophageal–gastric junction; KKRBB: Clinical Cancer Registry for Brandenburg and Berlin; DGC: diffuse type gastric cancer; FIGC: familial intestinal gastric cancer; GC: gastric cancer; HDGC: hereditary diffuse gastric cancer; UICC: union internationale contre le cancer.
