# Supplementary material for: Frequency of Positive Familial Criteria in Patients with Adenocarcinoma of the Esophageal-Gastric Junction and Stomach: First Prospective Data in a Caucasian Cohort
Source: Cancers (Basel). 2022 Jul 23;14(15):3590. doi: 10.3390/cancers14153590 (PMC9330468; doi:10.3390/cancers14153590)
Supplement: Supplementary file 1 [file cancers-14-03590-s001.zip › cancers-1744973-supplementary.pdf]

Table S1. Comparison of EpihiB (Epidemiology of hereditary gastric cancer in Berlin) data with data of the Clinical Cancer Registry for Brandenburg and Berlin (KKRBB) (AEG: adenocarcinoma of the esophageal–gastric junction; UICC: union internationale contre le cancer).

**header**	**All**					**Gastric Cancer**					**AEG**				
**header**	**KKRBB**		**EpiHib**			**KKRBB**		**EpiHib**			**KKRBB**		**EpiHib**		
**header**	n	(%)	n	(%)	p	n	(%)	n	(%)	p	n	(%)	n	(%)	p
Sex															
Female	207	(35.6)	56	(36.6)	0.823	161	(43.2)	42	(45.7)	0.666	46	(22.1)	14	(23)	0.863
Male	374	(64.4)	97	(63.4)		212	(56.8)	50	(54.3)		162	(77.9)	47	(77.0)	
Age Decades															
<20	0	(0)	2	(1,3)	**0.017**	0	(0.0)	2	(2.2)	**0.047**	0	(0.0)	0	(0.0)	0.092
20–29	4	(0.7)	2	(1.3)		4	(1.1)	2	(2.2)		0	(0.0)	0	(0.0)	
30–39	7	(1.2)	5	(3.3)		5	(1.3)	2	(2.2)		2	(1.0)	3	(4.9)	
40–49	23	(4)	9	(5.9)		17	(4.6)	7	(7.6)		6	(2.9)	2	(3.3)	
50–59	90	(15.5)	23	(15.0)		56	(15.0)	19	(20.7)		34	(16.3)	4	(6.6)	
60–69	134	(23.1)	44	(28.8)		76	(20.4)	21	(22.8)		58	(27.9)	23	(37.7)	
70–79	195	(33.6)	47	(30.7)		130	(34.9)	26	(28.3)		65	(31.3)	21	(34.4)	
80–89	111	(19.1)	19	(12.4)		72	(19.3)	11	(12)		39	(18.8)	8	(13.1)	
90–99	17	(2.9)	2	(1.3		13	(3.5)	2	(2.2)		4	(1.9)	0	(0.0)	
Localization															
AEG	208	(35.8)	61	(39.9)	0.353										
Stomach	373	(64.2)	92	(60.1)											
UICC Stage															
I	83	(14.3)	14	(9.2)	0.197	50	(13.4)	8	(8.7)	0.762	33	(15.9)	6	(9.8)	0.105

II	98	(16.9)	23	(15.0)		67	(18.0)	16	(17.4)		31	(14.9)	7	(11.5)
III	125	(21.5)	31	(20.3)		71	(19.0)	17	(18.5)		54	(26.0)	14	(23.0)
IV	171	(29.4)	59	(38.6)		125	(33.5)	35	(38.0)		46	(22.1)	24	(39.3)
Unknown	104	(17.9)	26	(17)		60	(16.1)	16	(17.4)		44	(21.2	10	(16.4)
All	581	(100)	153	(100)		373	(100)	92	(100)		208	(100)	61	(100)
